# The Effect of Antenatal Depression and Selective Serotonin Reuptake Inhibitor Treatment on Nerve Growth Factor Signaling in Human Placenta

**DOI:** 10.1371/journal.pone.0116459

**Published:** 2015-01-22

**Authors:** Helena Kaihola, Jocelien Olivier, Inger Sundström Poromaa, Helena Åkerud

**Affiliations:** 1 Department of Women’s and Children’s Health, Uppsala University, Uppsala, Sweden; 2 Department of Behavioural Physiology, University of Groningen, Groningen, The Netherlands; Radboud University, NETHERLANDS

## Abstract

Depressive symptoms during pregnancy are common and may have impact on the developing child. Selective serotonin reuptake inhibitors (SSRIs) are the most prescribed antidepressant treatment, but unfortunately, these treatments can also negatively affect the behavioral development and health of a child during pregnancy. In addition, serotonin (5-HT) exerts neurotrophic actions with thus far not fully known effects in the offspring. The neurotrophic growth factor (NGF) is involved in neuronal cell survival and differentiation, and altered placenta levels have been found to increase the risk for pregnancy complications, similar to those found in women treated with SSRIs. We therefore investigated whether the NGF signaling pathway was altered in the placenta from women treated with SSRIs (n = 12) and compared them with placenta from depressed (n = 12) and healthy mothers (n = 12). Results from immunohistochemical stainings revealed that placental NGF protein levels of SSRI-treated women were increased in both trophoblasts and endothelial cells compared with depressed and control women. In addition, downstream of the NGF receptor TrkA, increased levels of the signaling proteins ROCK2 and phosphorylated Raf-1 were found in stromal cells and a tendency towards increased levels of ROCK2 in trophoblasts and endothelial cells in SSRI-treated women when compared to healthy controls. SSRI-treated women also displayed increased levels of phosphorylated ROCK2 in all placental cell types studied in comparison with depressed and control women. Interestingly, in placental endothelial cells from depressed women, NGF levels were significantly lower compared to control women, but ROCK2 levels were increased compared with control and SSRI-treated women. Taken together, these results show that the NGF signaling and downstream pathways in the placenta are affected by SSRI treatment and/or antenatal depression. This might lead to an altered placental function, although the clinical relevance of our findings still needs to be investigated.

## Introduction

Almost 20% of women suffer from depressive symptoms during pregnancy and 4–7% are diagnosed with major depressive disorders [1–4]. When antidepressant treatment is needed during pregnancy, selective serotonin reuptake inhibitors (SSRIs) are the most widely prescribed as they are considered to be efficient, safe and have relatively few side-effects [5–7]. Currently, around 2–3% of the women in Europe are using antidepressants during pregnancy [8,9]. However, SSRIs have been shown to cross the placenta and are found in the amniotic fluid and cord blood [10–13]. SSRI treatment during pregnancy has been associated with an increased risk of poor pregnancy outcomes including premature birth, impaired fetal placental function and decreased fetal body and head growth, but these outcomes are also found in offspring of mothers with antenatal depression (reviewed by [14–17]). Similarly, it has been shown that SSRI treatment as well as antenatal depression can cause behavioral disorders (reviewed in [14–17]), why it remains unclear which effects are caused by the antenatal depression *per se* and what is caused by the pharmacological treatment of the depression.

Serotonin (5-HT) acts as a neurotrophic factor during brain development, indicating that alterations in 5-HT levels due to SSRI treatment might affect neurodevelopment, e.g. cell division, differentiation and dendritic pruning [18,19]. Recently a placental 5-HT synthetic pathway was discovered [20], and in addition, the 5-HT produced by the placenta was selectively accumulated in the fetal forebrain during the initial axon growth period [20], ultimately suggesting that serotonergic agents may have indirect effects on fetal development. 5-HT may also influence placental function, which in turn, may have consequences for the fetus. For instance, 5-HT is a key regulator of embryogenesis [21] and placentation [22], and also acts as a powerful vasoconstrictor agent in the placenta [23]. As such, 5-HT has been implicated in preeclampsia [24] and gestational diabetes pathophysiology [25]. Furthermore, maternal SSRI treatment has been shown to alter the placental barrier, via increased multidrug resistance phosphoglycoprotein (P-gp)-mediated substrate efflux [26], but these drugs have no effect beyond that of depression and/or anxiety on monoamine transporter gene expression [27].

NGF has a role in neuronal cell survival and differentiation, as well as in non-neuronal processes e.g. immunomodulation, angiogenesis and folliculogenesis [28–30], and is expressed in a number of tissues, including the placenta [31]. In the placenta, NGF is involved in placentation [32] and pregnancy maintenance [28], and has thus been implicated in stress-induced miscarriage [33] and preterm birth [34]. Also, increased production of NGF has been shown to reduce fertility in mice [35]. Notably, some of these outcomes are also among those reported to be more frequent in SSRI-treated pregnant women [36].

**Figure 1:**
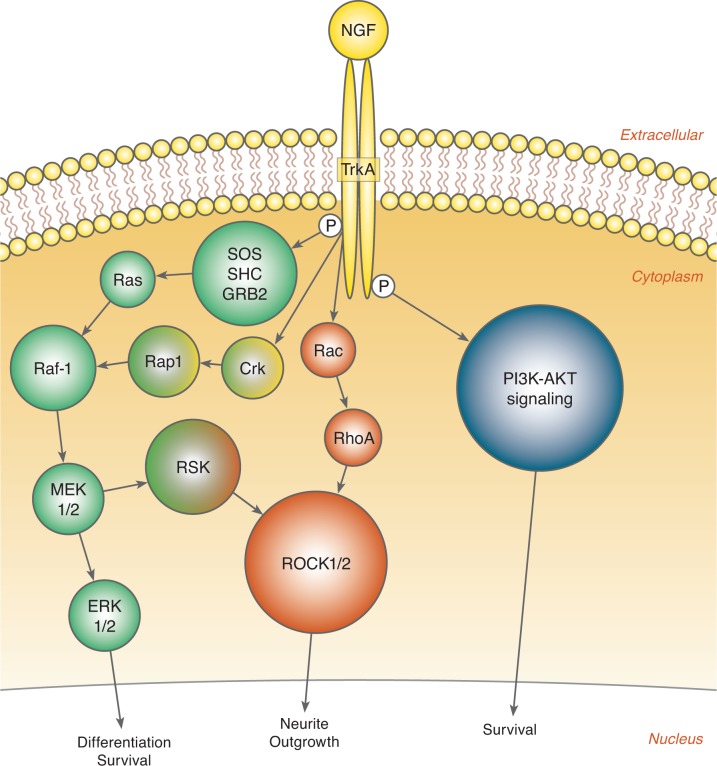
Simplified schematic figure of the signaling pathways down-stream of NGF and its receptor TrkA.

Upon binding to the TrkA receptor, NGF induces autophosphorylation on different tyrosine residues. A dimerized TrkA receptor is formed and several signaling cascades are initiated, e.g. the Ras-Raf-MAPK pathway [37] (Fig. 1). Downstream of this pathway, ROCK can be activated both via the Rac-RhoA or the Raf-MAPK-RSK signaling pathway [38–41].

Based on the limited knowledge on how SSRI treatment affects placental function, the aim of the present study was to investigate the independent influence of SSRI treatment and antenatal depression on placental proteins in the NGF signaling pathway (Fig. 1). We hypothesized that maternal SSRI use would alter NGF signaling and downstream pathways in the placenta.

## Materials and Methods

### Study population material

This study was carried out at the Department of Women’s and Children’s health, Uppsala University Hospital, as a sub-study within an ongoing longitudinal study on antenatal and postpartum depression: the ‘Biology, Affect, Stress, Imaging and Cognition in Pregnancy and the Puerperium’ (BASIC) study. The BASIC study and this sub-study were approved by the Regional Ethics Committee, Uppsala, Sweden (approval number 2009/171). All women attending the routine ultrasound at gestational week 16-18 at Uppsala University Hospital are approached for participation in the study, enabling a population-based sampling. To date, the BASIC study has included 3,800 women. Oral and written information about the study objectives and about collection of biological samples, including placental tissue at delivery (sub-study open between February 2010 and March 2012), was given and informed written consent was obtained. Exclusion criteria for the BASIC study were (1) inability to adequately communicate in Swedish, (2) women whose personal data were kept confidential, (3) women with pathologic pregnancies as diagnosed by routine ultrasound, and (4) women younger than 18 years. Women were asked to fill out a web-based questionnaire containing the Swedish version of the Edinburgh Postnatal Depression Scale (EPDS). Both questionnaires have been validated for use in both pregnant and postpartum women [42]. Depressive symptoms were scored in gestational week 17 and gestational week 32. In addition, the questionnaires also included questions on physical and socio-demographic characteristics, medical, psychiatric, gynecologic and obstetric history variables, lifestyle, and medication parameters. Information concerning the antenatal depression, SSRI use, clinical variables, delivery and neonatal outcomes were retrieved from the medical records.

For the entire BASIC-placenta sub-study, 913 women with placenta samples were available. For this sub-study three different groups of women were included; healthy pregnant controls (n = 12), depressed pregnant women (n = 12) and SSRI-treated pregnant women (n = 12). To allow for as comparable groups as possible, without any interference of other placental disturbances, an additional set of inclusion and exclusion criteria was applied. Inclusion criteria were women of Western European descent, normal pregnancies, normal deliveries, and healthy offspring (no diagnoses and no admittance to neonatal care). Exclusion criteria for all groups were smoking or alcohol use during pregnancy, any daily use of prescribed drugs during pregnancy, any other chronic conditions or diseases, gestational age < 35 weeks, and maternal age < 18 or > 42 years. Depressed cases had EPDS scores of 13 or higher in gestational weeks 17 *and* 32 indicative of antenatal depression, together with hospital records confirming major or minor depressive disorder and ongoing treatment for their depression in terms of psychotherapy. Women on SSRI treatment had used their treatment during the entire pregnancy in clinically relevant doses, i.e. low-dose use was excluded. The groups were matched on maternal age.

### Placental tissue collection

Placental tissue biopsies (all the way through the placenta, which means from the fetal to maternal side) were obtained at delivery from two different representative locations on the placenta, rinsed in cold sterile phosphate-buffered saline to wash off maternal and fetal blood, and snap frozen on dry ice. Tissue pieces were frozen within 60 minutes after delivery and stored at −70°C until further use.

### Protein isolation

Protein extract was prepared from frozen placenta. Pieces were collected from the fetal side of the placenta and the procedure was performed by the same person for all preparations. The protein isolation was performed using a RIPA lysis buffer (catalogue no R0278, Sigma-Aldrich Corp., US) containing Protease Inhibitor cocktail (catalogue no P8340, Sigma-Aldrich Corp., US), 1 mM phenylmethylsulfonyl fluoride (PMSF) and 1 mM ortovanadate. All biopsies were homogenized, incubated and centrifuged. The supernatant containing the total lysate was collected and protein concentration was measured at 595 nm on a spectrophotometer using Bradford reagent (catalogue no B6916, Sigma-Aldrich Corp., US). Every protein lysate was separated on a NuPage Novex 4-16% Bis-Tris gel (catalogue no NP0321) with MOPS SDS Running buffer (catalogue no NP0001) (both from Invitrogen, Life technologies, Thermo Scientific Inc., US). The separated proteins were transferred to an Immobilon-FL PVDF membrane (catalogue no IPFL00010, Merck Millipore, US) using wet transfer.

### Western blot

After blocking with Odyssey Blocking buffer (catalogue no 927-40000, LI-COR Biosciences Inc., US), the membranes were incubated for 1 hour at room temperature (RT) or overnight at 4°C with protein-specific primary antibodies against NGF (catalogue no sc-548), Raf-1 (catalogue no sc-133), RhoA (catalogue no sc-418), ROCK1 (catalogue no sc-5560), ROCK2 (catalogue no sc-1851), and p-TrkA (Tyr 496) (catalogue no sc-7987-R), all from SantaCruz Biotechnology Inc., US. Antibodies against TrkA (catalogue no 06-574, Upstate, Merck Millipore, US) and phosphorylated TrkA (p-Y490) (catalogue no ab1445, Abcam plc, England) were also used. All protein levels were normalized against β-actin, detected by a primary antibody from SantaCruz Biotechnology Inc., US (catalogue no sc-47778). For dilutions of the primary antibodies, see Table 1. After incubation, the membranes were washed with Tris-buffered saline (TBS)-0.1% Tween 3 × 10 minutes on a seesaw, followed by incubation for 1 hour at RT with secondary antibodies labeled with fluorophores IRDye 800CW or IRDye 680RD (LI-COR Biosciences Inc., US). The secondary antibodies were all diluted 1:10,000. All primary and secondary antibodies were diluted in Odyssey Blocking buffer. After incubation with the secondary antibodies, the membranes were washed 3 × 10 minutes with TBS-0.1%Tween on a seesaw and the proteins detected at 700nm and 800nm using an Odyssey Image scanner (LI-COR Biosciences Inc., US).

**Table 1:** Clonality, host species and dilutions of antibodies used in Western blot analysis and immunohistochemistry.

**Target**	**Catalogue no**	**Commercial supplier**	**Clonality**	**Host species**	**Dilution for WB**	**Dilution for IHC**
NGF	sc-548	SantaCruz Biotechnology Inc., US	polyclonal	rabbit	1:200	1:500
TrkA	06-574	Upstate, Merck Millipore, US	polyclonal	rabbit	1:500	1:100
pTrkA (pTyr490)	ab1445	Abcam plc, England	polyclonal	rabbit	1:500	N/A
pTrkA (pTyr496)	sc-7987-R	SantaCruz Biotechnology Inc., US	polyclonal	rabbit	1:200	N/A
Raf-1	sc-133	SantaCruz Biotechnology Inc., US	polyclonal	rabbit	1:1000	1:200
pRaf-1 (pTyr340/341)	sc-16806	SantaCruz Biotechnology Inc., US	polyclonal	goat	N/A	1:200
RhoA	sc-418	SantaCruz Biotechnology Inc., US	monoclonal	mouse	1:1000	1:500
ROCK1	sc-5560	SantaCruz Biotechnology Inc., US	polyclonal	rabbit	1:1000	1:200
ROCK2	sc-1851	SantaCruz Biotechnology Inc., US	polyclonal	goat	1:1000	1:200
pROCK2 (pSer1366)	PA5-34895	Thermo Scientific Inc., US	polyclonal	rabbit	N/A	1:100
β-Actin	sc-47778	SantaCruz Biotechnology Inc., US	monoclonal	mouse	1:1000	N/A

WB = Western blot, IHC = Immunohistochemistry

### Immunohistochemistry

Paraffin-embedded placental biopsies were sectioned into 5 μm thin slices, mounted on glass slides and dried at 37°C for at least overnight. The sections were deparaffinized in xylene, rehydrated in different ethanol concentrations (3 minutes in 99.5%, 3 minutes in 95% and 3 minutes in 70%) and washed once in deionized water and 2 × 5 minutes in Phosphate buffered saline (PBS), pH 7.4. Antigenic retrieval was performed by heating the slides in 0.01M citrate buffer, pH 6.0, in a water bath in a microwave oven for 10 minutes at 650W. The slides were allowed to cool down at RT were then washed 3 × 5 minutes in PBS, fixated in ice-cold acetone/methanol mix (1:1) for 15 minutes in RT, washed 3 × 5 minutes in PBS, incubated against endogenous peroxidase activity with 3% H_2_O_2_ in methanol for 10 minutes and then washed again 3 × 5 minutes in PBS. Non-specific binding was blocked by incubating the sections in sterile PBS containing 5% horse (catalogue no S-2000, Vector Laboratories Inc., US) or goat serum (catalogue no X0907, Dako, Denmark) for 1 hour at RT in a humidified chamber. The sections were incubated with a primary antibody diluted in 0.1% BSA-PBS overnight in a moist chamber at 4°C. Primary antibodies were against NGF (catalogue no sc-548), Raf-1 (catalogue no sc-133), p-Raf-1 (Tyr 340/341) (catalogue no sc-16806), RhoA (catalogue no sc-418), ROCK1 (catalogue no sc-5560), ROCK2 (catalogue no sc-1851), all from SantaCruz Biotechnology Inc.,US. Also primary antibodies against TrkA (catalogue no 06-574, Upstate, Merck Millipore, US) and p-ROCK2 (Ser1366) (catalogue no PA5-34895, Thermo Scientific Inc., US) were used. For dilutions of primary antibodies, see Table 1. After washing the slides with PBS-0.1% Tween20 for 3 × 5 minutes, the sections were incubated with biotinylated horse-anti-goat (catalogue no BA-9500), horse-anti-mouse (catalogue no BA-2000) or goat-anti-rabbit (catalogue no BA-1000) antibodies (all from Vector Laboratories Inc., US) diluted 1:300 in 0.1% BSA-PBS for 1 hour in RT. The sections were washed 3 × 5 minutes with PBS-0.1% Tween 20 and incubated with avidinD conjugated horseradish peroxidase (catalogue no X0408, Vector Laboratories Inc., US) diluted 1:400 in sterile PBS for 1 hour in RT in a moist chamber. After washing the slides with PBS-0.1% Tween20 for 3 × 5 minutes, the sections were stained/developed using liquid 3,3´-diaminobenzidine tetrahydrochloride (DAB) + substrate chromogen system (catalogue no K3468, Dako, Denmark), washed for 5 minutes in running tap water, counterstained with Mayer hematoxylin (catalogue no 01820, HistoLab Products, Sweden) and rinsed under running tap water for 5 minutes. The sections were dehydrated in deionized water for 30 seconds, different ethanol concentrations (3 minutes in 70%, 3 minutes in 95% and 3 minutes in 99.5%) and 2 × 5 minutes in xylene, and mounted under cover glasses.

Scoring based on staining intensity was done by visual inspection in a light microscope (40x objective; Axio Observer.Z1, Carl Zeiss AG Corp. Germany). A scale with the range 1 to 4 was used, where 1 corresponds to the lowest intensity and 4 the highest. A double-blinded validation of the scoring was performed where a second person scored the slides in the same way as the first one without knowing the initial results.

### Statistical methods

Clinical characteristics were compared by Pearson Chi-Square test or medians using Mann-Whitney *U* test. Differences in protein expression detected by Western blot (WB) or immunohistochemistry were evaluated by use of Mann-Whitney *U* test. Level of significance was set at p < 0.05. Data were analyzed using the Statistical Package for the Social Sciences 20.0 software (SPSS Inc, Chicago, IL, USA) for windows.

## Results

For the entire BASIC-placenta sub-study, 913 women with placenta samples were available. Of these, 115 (12.6%) had elevated depression scores at some point during pregnancy. The 12 depressed women included in this study did not differ in terms of age, BMI, birth weight or gestational length in comparison with the remaining depressed women (data not shown), but had higher median depression scores at gestational week 17 (17.0 vs. 13.0, p < 0.05) and 32 (16.0 vs. 14.0, p < 0.05). Similarly, 43 (4.7%) women in the entire BASIC placenta sub-study used SSRI during pregnancy. The 12 SSRI users included in this study did not differ in terms of age, BMI, birth weight or gestational length in comparison with the remaining SSRI-treated women (data not shown), but had slightly, but not significantly, higher median depression scores at gestational week 17 (10.5 vs. 7.0) and 32 (11.5 vs. 7.0).

Demographic data of the study population are displayed in Table 2. For data on each individual woman, see S1 Table. SSRI-treated women and depressed women were more often parous than healthy controls, and SSRI-treated women had significantly shorter gestational length than healthy controls (Table 2). SSRI-treated women also had higher BMI than depressed women, but did not differ from controls. As expected, both SSRI-treated and depressed women had higher self-rated depression scores in gestational week 17 and 32 compared with healthy controls. However, self-rated depression scores were significantly lower in the SSRI-treated women than in the depressed women (Table 2). Otherwise, no significant differences between groups in age, blood pressure in late pregnancy, smoking frequency, number of IVF-treated women, birth weight or sex of the child were noted.

**Table 2:** Demographic data of the study groups.

	**Controls n = 12**	**Depressed n = 12**	**SSRI-treated n = 12**
**Age, year**	28.5 (25.0–33.0)	31.5 (26.0–36.0)	29.0 (25.0–35.0)
**Parous women, n (%)**	3 (25.0%)	10 (83.3%)*	8 (66.7%)*
**BMI**	24.2 (20.1–31.7)	24.3 (18.2–45.4)	27.0 (22.7–35.8)^a^
**MAP first trimester**	97.5 (82.5–112.0)	85.2 (77.5–104.0)*	93.0 (85.0–102.0)
**MAP partus**	103.8 (87.5–120.0)	99.5 (89.5–110.0)	101.2 (94.0–113.0)
**Smokers, n (%)**	0	1 (8.3%)	2 (16.7%)
**IVF treatment**	0	0	1 (8.3%)
**Gestational length, days**	282 (272–289)	277 (263–293)	272 (263–284)**
**Sex of child, n of girls/boys (%)**	5 / 7 (41.7% / 58.3%)	5 / 7 (41.7% / 58.3%)	8 / 4 (66.7% / 33.3%)
**Birth weight, grams**	3520 (3110–4080)	3730 (2890–4540)	3510 (3170–4230)
**EPDS gestational week 17**	3.0 (0.0–5.0)	17.0 (4.0–24.0)***	10.5 (0.0–20.0)**, ^a^
**EPDS gestational week 32**	3.5 (1.0–6.0)	16.0 (11.0–23.0)***	11.5 (3.0–24.0)***, ^a^

Blood pressure is shown as mean arterial pressure (MAP). Data are presented as median (minimum–maximum).

* p < 0.05, significantly different in comparison with controls, Pearson Chi-Square test

** p < 0.01, significantly different in comparison with controls, Mann-Whitney *U* test

*** p < 0.001, significantly different in comparison with controls, Mann-Whitney *U* test

^a^p < 0.05, significantly different compared to depressed, Mann-Whitney *U* test

### Western blot

In placental total lysates no differences were found in NGF, TrkA, phosphorylated TrkA (both pY490 and pY496), Raf-1, RhoA, ROCK1 and ROCK2 protein levels between SSRI-treated women, depressed women and healthy controls (Table 3).

**Table 3:** Placental protein levels detected by Western blot.

	**Controls n = 12**	**Depressed n = 12**	**SSRI-treated n = 12**
**NGF**	0.18 (0.01–0.79)	0.15 (0.02–1.12)	0.30 (0.01–1.00)
**TrkA**	0.05 (0.01–0.14)	0.04 (0.01–0.14)	0.02 (0.01–0.18)
**TrkA pY490**	0.02 (0.01–0.08)	0.02 (0.01–0.10)	0.02 (0.00–0.09)
**TrkA pY496**	0.44 (0.30–0.85)	0.51 (0.12–2.52)	0.38 (0.12–1.55)
**Raf-1**	0.55 (0.22–1.04)	0.61 (0.23–1.26)	0.65 (0.28–0.94)
**RhoA**	0.05 (0.02–0.13)	0.04 (0.02–0.16)	0.06 (0.02–0.14)
**ROCK1**	1.19 (0.74–3.60)	1.39 (0.40–2.52)	1.19 (0.64–2.97)
**ROCK2**	1.37 (0.47–4.69)	2.15 (0.68–4.18)	2.26 (0.94–4.46)

Data are presented as median (minimum–maximum). No significant differences were found between groups, Mann-Whitney *U* test.

### Immunohistochemistry

**Figure 2:**
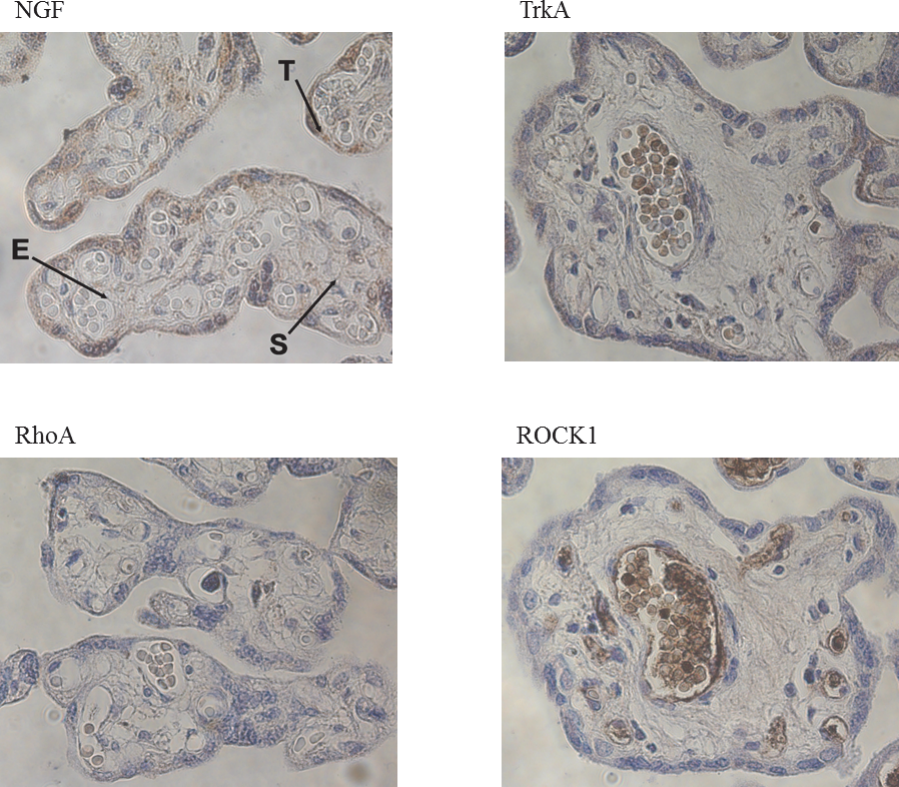
Immunohistochemical stainings of placenta. All stainings are from healthy control women. T = trophoblasts, E = endothelial cells, S = stromal cells.

**Figure 3:**
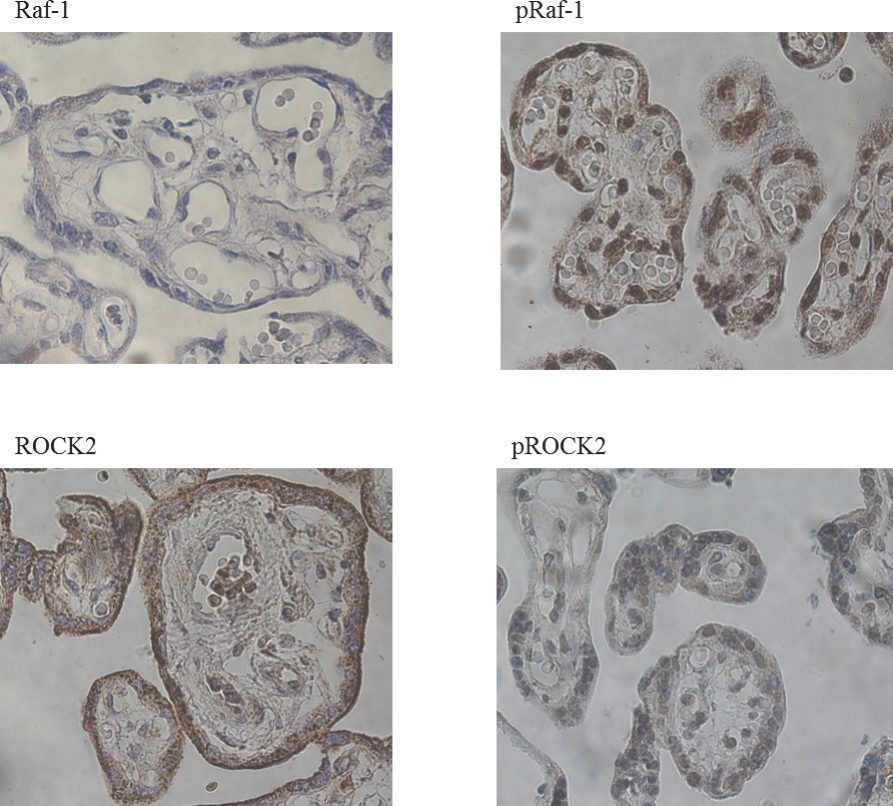
Immunohistochemical stainings for Raf-1, phosphorylated Raf-1 (pRaf-1), ROCK2 and phosphorylated ROCK2 (pROCK2) in placenta. All stainings are from healthy control women.

Immunohistochemical staining of placental biopsies indicated that NGF, TrkA, RhoA, Raf-1, phosphorylated Raf-1, ROCK2, and phosphorylated ROCK2 were found at high levels in the trophoblasts, less in the endothelial cells, and at very low levels in the stromal cells (Figs. 2 and 3). ROCK1, on the other hand, had a different staining pattern with the highest levels in the endothelial cells, lower levels in the trophoblasts, and the weakest staining in stromal cells. As an indicative of active signaling, phosphorylated Raf-1 and phosphorylated ROCK2 had the highest staining intensity in the nucleus, whereas staining for total Raf-1 and ROCK2 were seen both in the nucleus and cytoplasm (Fig. 3).

**Figure 4:**
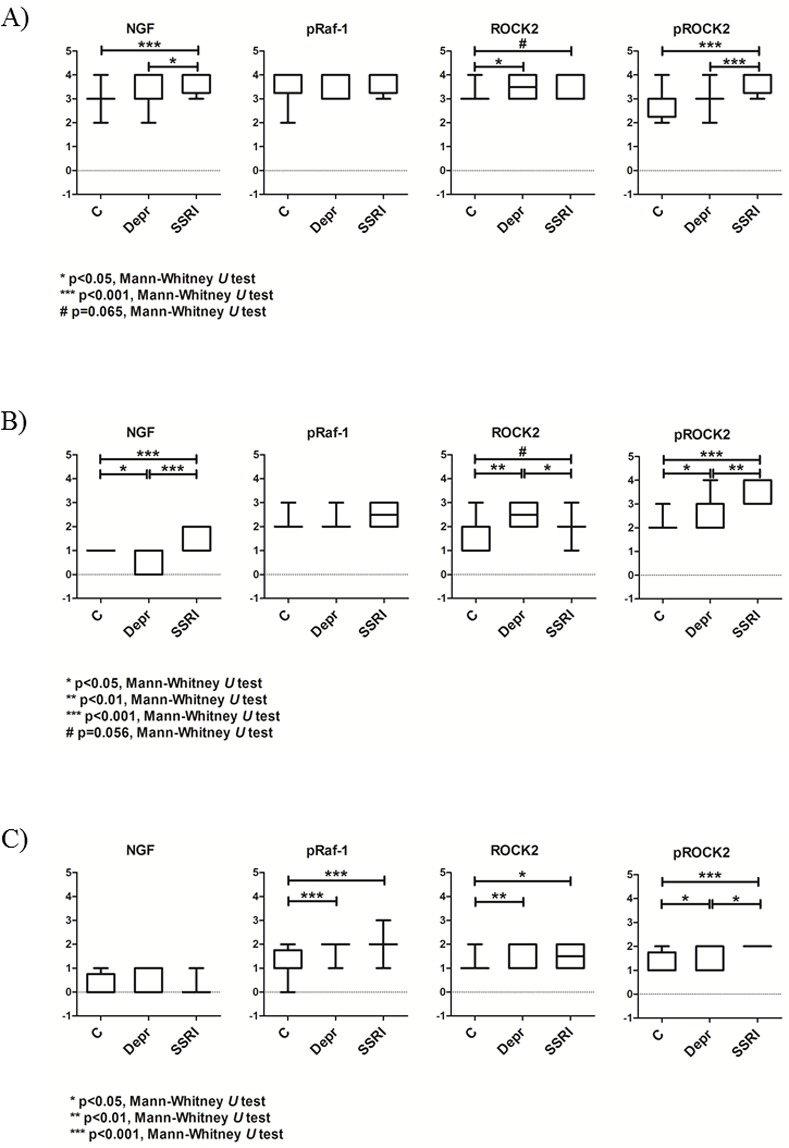
Protein levels in different cell types of placenta detected by immunohistochemistry. Placental sections stained for NGF, phosphorylated Raf-1 (pRaf-1) and ROCK2 in A) Trophoblasts, B) Endothelial cells and C) Stromal cells.

While no difference in protein levels of the placental total lysates was detected between SSRI-treated women, depressed women and controls, the immunohistochemical staining, where the different cell types of the placenta were considered separately, clearly suggested altered protein levels between groups. The placental NGF staining intensity was increased in SSRI-treated women compared with depressed women and healthy controls in both trophoblasts (p < 0.05 and p < 0.001, respectively) and endothelial cells (p < 0.001 and p < 0.001, respectively) (Fig. 4A and 4B). The placental NGF staining intensity in endothelial cells was decreased in depressed women compared with controls (p< 0.05, Fig. 4B). No difference in NGF staining intensity in stromal cells was noted between groups (Fig. 4C).

The level of phosphorylated Raf-1 was increased in stromal cells of both SSRI-treated women and depressed women in comparison with healthy controls (p < 0.001 and p < 0.001, respectively; Fig. 4C). No difference in phosphorylated Raf-1 staining intensity was found between groups in the trophoblasts or the endothelial cells (Fig. 4A and 4B).

There was a tendency towards increased ROCK2 levels in SSRI-treated women in comparison with controls in trophoblasts (p = 0.065) and endothelial cells (p = 0.056) (Fig. 4A and 4B). ROCK2 levels in stromal cells of SSRI-treated women were higher than in the controls (p < 0.05). However, ROCK2 levels in placental endothelial cells were significantly lower in SSRI-treated women than in depressed women (p < 0.05, Fig. 4B). In addition, depressed women had increased ROCK2 staining intensity in comparison with controls in all cell types (trophoblasts p < 0.05, endothelial cells p < 0.01, and stromal cells p < 0.01) (Fig. 4A–C).

The placental staining intensity of phosphorylated ROCK2 was increased in SSRI-treated women compared with depressed women and healthy controls in trophoblasts (p < 0.001 and p < 0.001, respectively), endothelial cells (p < 0.01 and p < 0.001, respectively), and stromal cells (p < 0.05 and p < 0.001, respectively) (Fig. 4A–C). In addition, the staining intensities of phosphorylated ROCK2 in endothelial and stromal cells were higher in depressed women than in healthy controls (p < 0.05 and p < 0.05, respectively) (Fig. 4B and 4C).

No significant differences in staining intensities of TrkA, Raf-1, RhoA and ROCK1 were found between groups in placental trophoblasts, endothelial or stromal cells (S1A–C Fig.).

## Discussion

In this study we have shown that the NGF signaling pathway is altered in the placenta of SSRI-treated women in comparison with depressed women and healthy controls. Placental NGF was increased in both trophoblasts and endothelial cells of SSRI-treated women. Downstream of the TrkA receptor, SSRI-treated women displayed increased phosphorylated Raf-1 levels in stromal cells, increased levels of ROCK2 in stromal cells, a tendency towards increased levels of ROCK2 in trophoblasts and endothelial cells, and increased levels of phosphorylated ROCK2 in all placenta cell types studied, and these differences were evident in comparison with healthy women. Compared with depressed women, SSRI-treated women displayed decreased ROCK2 levels in placental endothelial cells and increased phosphorylated ROCK2 levels in trophoblasts, endothelial and stromal cells.

In placental tissue of depressed women, on the other hand, lower NGF levels in endothelial cells were found not only in comparison with the SSRI-treated women, but also in comparison with healthy controls. However, down-stream of the TrkA receptor, placental tissue of depressed women was characterized by increased phosphorylated Raf-1 in stromal cells, increased ROCK2 levels in all cell types, and increased phosphorylated ROCK2 in endothelial and stromal cells in comparison with controls. Thus, in comparison with healthy controls, the placental NGF signaling pathway is differently regulated in the SSRI-treated and the depressed women.

Although IHC revealed alterations in protein levels, this was not confirmed by WB analysis. This is most likely explained by the fact that in WB analysis all cell types in the placenta are pooled, meaning that a decrease in levels in one cell type might mask an increase in another cell type and *vice versa*, or a small effect in only one cell type might not be seen when all cell types are mixed together.

Until now few studies have described the SSRI-induced biological effects on placental function or the potential biological mechanisms that might explain why SSRI treatment is associated with an increased risk of developing maternal and fetal complications. Among these, an increased risk of miscarriage, premature birth, preeclampsia, together with a number of outcomes in the offspring including low birth weight and pulmonary hypertension have been reported (reviewed in [14–17]). The current study merely focused on effects of antenatal depression and SSRI use during pregnancy on NGF signaling. NGF has been shown to be expressed in human placenta [43,44] where it, in turn, is involved in important functions such as placentation [32] and pregnancy maintenance [28]. Thus, NGF signaling may have clinical relevance for miscarriage and preterm birth, as evidenced by animal and human studies [33,34,45]. Furthermore, adequate implantation/placentation is a prerequisite for a pregnancy to occur and a well-functioning placenta is of major importance for the intrauterine development and growth of a child [46,47]. Barker [48] hypothesized that diseases that might manifest later in life can be traced back to early development, and a mechanistic role of the placenta in fetal programming has been discussed during recent years [46]. This phenomenon is usually referred to as the Barker hypothesis and the relevance of this has also been confirmed by others [49–52]. Given the placental 5-HT synthesis [20] and the accumulation of placental 5-HT in the fetal forebrain during an important growth period [20], serotonergic agents may influence not only placental function, but also indirectly, fetal neurodevelopment. Our findings of differences in placental NGF signaling in SSRI-treated women add to the increasing literature on effects and consequences of SSRI use during pregnancy, although we at present are unable to speculate on whether this has any bearing on the offspring neurodevelopment.

Furthermore, a role for ROCK1 has been suggested in hypertension [53–56] and increased ROCK2 expression has been described in preeclamptic human placentas [57]. In the placenta, we have established that ROCK1 and ROCK2 are expressed in different cell types, with higher ROCK1 levels in the endothelial cells and ROCK2 predominantly found in the trophoblasts. Because inadequate trophoblast invasion and endothelial dysfunction are important features in the development of preeclampsia, and because SSRI-treated women had increased levels of ROCK2 in trophoblasts it may be speculated that NGF signaling also plays a role in preeclampsia. Indeed, SSRI-treated women have an increased risk of preeclampsia [58–60] and this risk also depends on duration of SSRI use during pregnancy, i.e. women using SSRIs during the entire pregnancy have an increased risk in comparison to those who discontinue before gestational week 20, and in comparison with non-users [58–60]. Finally, ROCK2 has been found in vascular smooth muscle cells and has shown to play a role in hypoxia-induced pulmonary hypertension in mice [61], yet another rare but important complication from SSRI use during pregnancy [8]. Although this study by no means is able to fully elucidate the role of NGF signaling for all of these maternal and fetal complications, our findings nevertheless points towards important distinctions between how the exposure to depression *per se* and SSRI treatment of depression affects placental function.

It has previously been reported that SSRI treatment increases the levels of Activin A in maternal blood, amniotic fluid and fetal cord blood [62]. Activin A acts as a neurotrophic factor and is known as a marker of brain-damage (reviewed in [63]). Belissima et al [62] suggested that the elevated levels of Activin A might indicate that antenatal SSRI treatment causes fetal brain-damage. However, in the study of Belissima and colleagues no comparison with untreated depressed women was performed which would have been of interest. There are furthermore, to our knowledge, no reports on cross-talk between NGF and Activin A signaling pathways. Another marker of importance might be Reelin, since it acts as a neurotrophic factor during development [64], and as altered Reelin levels have been shown to associate with psychiatric disorders, e.g. mood disorders or autism [65,66]. The relevance of Reelin in SSRI treatment is supported by a study suggesting decreased levels of Reelin in cord serum of SSRI-treated women compared to healthy controls [67]. These results are interesting since it has been shown that Reelin-deficient mice express lower levels of NGF [68] and the proteolysis of the ApoER2 receptor, after binding to its ligand Reelin, is regulated by NGF and also dependent on TrkA signaling [69]. The results that we present concerning the NGF signaling pathway in SSRI-treated women might be in agreement with this. When SSRI treatment increase levels of NGF in placenta, it might cause an increase in ApoER2 proteolysis, and thereby also an increase in signaling down-stream of the Reelin receptor.

A major problem in pharmaco-epidemiological studies on SSRI use during pregnancy is that these are unable to discriminate between effects caused by the antenatal depression *per se* and the effects induced by the treatment of the depression (i.e. SSRI). For instance, a number of studies have shown that SSRI treatment as well as antenatal depression can cause behavioral disorders (reviewed in [14]). Similarly, animal studies are also biased by the fact that SSRI use is merely compared to non-use in healthy, non-depressed animals, i.e. the effect of depression is not accounted for. Prenatal and postnatal SSRI treatment is associated with developmental alterations in rodent offspring (reviewed in [70,71]). So far, only two studies [72,73] have addressed the effects of developmental SSRI exposure on a maternal adversity model in rats. Importantly, they found that developmental fluoxetine exposure normalized long-term effects of maternal adversity on post-operative pain [73], and decreased the area of sexually dimorphic nucleus of the preoptic area in the brain [72]. Although these studies are discerning between fluoxetine treatment and maternal adversity, much more research addressing the effects of developmental antidepressant exposure in models of depression are necessary in order to unravel underlying mechanisms that are altered in offspring due to the drug treatment, the maternal adversity or to the combination.

In the present study, by incorporating a depressed group of women as additional controls to the SSRI users we are able to show additive effects by depression and SSRI use on certain aspects of NGF signaling (ROCK2 and phosphorylated ROCK2). Other parts of the signaling pathway, however, show opposing effects of depression *per se* and SSRI use, as in the case of NGF levels in endothelial cells. Even though our findings are not conclusive in terms of clinical relevance, our results contribute to the overall understanding of the complexity.

In conclusion, we found that SSRIs interact with NGF signaling in placenta and SSRI seems furthermore to affect levels of proteins in the NGF signaling pathway, mainly NGF, phosphorylated Raf-1, ROCK2, and phosphorylated ROCK2. This might affect placental function and possibly the intrauterine development of the child. The exact mechanism and clinical relevance of this is still not clear and needs to be investigated further in the future.

## Supporting Information

S1 Table Data of the women included in this study. (XLSX)

S1 Fig Protein levels in different cell types of placenta detected by immunohistochemistry. Placental sections stained for TrkA, Raf-1, RhoA and ROCK1 in A) Trophoblasts, B) Endothelial cells and C) Stromal cells.
(TIF)
