# Supplementary figures and images for: The Effect of Antenatal Depression and Selective Serotonin Reuptake Inhibitor Treatment on Nerve Growth Factor Signaling in Human Placenta
Source: PLoS One. 2015 Jan 22;10(1):e0116459. doi: 10.1371/journal.pone.0116459 (PMC4303267; doi:10.1371/journal.pone.0116459)

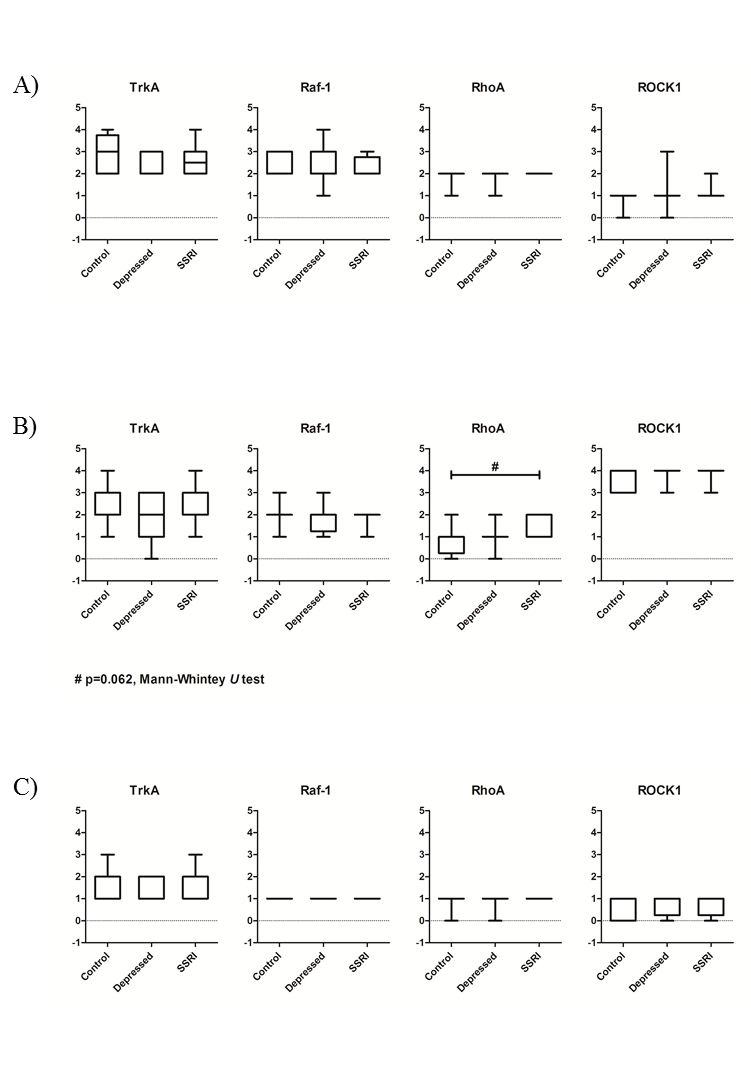

Supplement: S1 Fig — Placental sections stained for TrkA, Raf-1, RhoA and ROCK1 in A) Trophoblasts, B) Endothelial cells and C) Stromal cells. (TIF) [file pone.0116459.s002.tif]
